# A New Cembrane Diterpene from the Bornean Soft Coral *Nephthea* sp

**DOI:** 10.3390/molecules15063857

**Published:** 2010-05-26

**Authors:** Takahiro Ishii, Zhan Zhaoqi, Charles Santhanaraju Vairappan

**Affiliations:** 1 Laboratory of Natural Products Chemistry, Institute for Tropical Biology and Conservation, Universiti Malaysia Sabah, 88999 Kota Kinabalu, Sabah, Malaysia; E-Mail: ishii_t@ums.edu.my (T.I.); 2 Shimadzu (Asia Pacific) Pte Ltd, 16 Science Park Drive, #01-01, The Pasteur Singapore Science Park, Singapore 118227, Singapore; E-Mail: zhaoqi@shimadzu.com.sg (Z.Z.)

**Keywords:** cembrane, diterpene, *Nephthea* sp., Nephtheidae, soft coral

## Abstract

A new cembrane diterpene, 6-acetoxy-7,8-epoxynephthenol acetate (**1**) was isolated along with a known compound, epoxynephthenol acetate (**2**), from the organic extract of a Bornean soft coral *Nephthea* sp. Their structures were elucidated on the basis of spectroscopic analyses and comparison with those previous literature data.

## 1. Introduction

**Figure 1:**
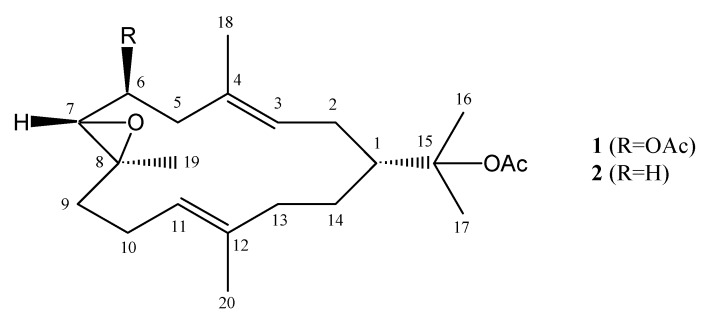
Structures of compounds **1** and **2**.

Soft corals belonging to the genus *Nephthea* (Alcyonacea, Nephtheidae) are a rich source of sesquiterpenoids, diterpenoids and steroids with diverse chemical structures and interesting biological activities [1,2,3,4]. Our previous chemical investigations on the Bornean soft coral genus *Nephthea* have resulted in the isolation and identification of a new sterol [5] and a new norsesquiterpenoid [6], along with several known sesquiterpenes [6]. However, to date no diterpenes have been isolated from these Bornean soft corals. In the course of our interest in the discovery of other types of secondary metabolites from this genus, we examined a specimen collected from Layangan Island (Sabah, Malaysia). The methanol extract afforded a new cembrane diterpene, 6-acetoxy-7,8-epoxynephthenol acetate (**1**), along with a known compound, epoxynephthenol acetate (**2**) [3,7,8,9] (Figure 1). This paper reports on the isolation and structure elucidation of the new compound.

## 2. Results and Discussion

Compound **1** was isolated as a colorless oil. HR-MS gave a molecular formula of C_24_H_38_O_5_ with six degrees of unsaturation. The ^1^H- and ^13^C-NMR spectral data (Table 1) revealed the presence of an acetoxy group [δ_C_ 170.3 (s), 21.2 (q); δ_H_ 2.08 (3H, s)], an acetoxyisopropyl residue [δ_C_ 170.2 (s), 85.6 (s), 23.2 (q), 23.2 (q), 22.6 (q); δ_H _1.97 (3H, s), 1.46 (3H, s), 1.44 (3H, s)], an oxymethine [δ_C_ 71.4 (d); δ_H _4.91 (1H, ddd, *J* = 8.0, 7.5, 3.0 Hz)], a trisubstituted epoxide [δ_C_ 63.3 (d), 61.2 (s); δ_H _3.04 (1H, d, *J* = 8.0 Hz)], two trisubstituted double bonds [δ_C_ 135.0 (s), 130.3 (d), 128.1 (s), 125.1 (d); δ_H _5.49 (1H, dd, *J* = 7.4, 7.4 Hz), 5.13 (1H, dd, *J* = 7.0, 7.0 Hz)], and three tertiary methyls [δ_C_ 17.4 (q), 17.1 (q), 15.0 (q); δ_H _1.71 (3H, s), 1.56 (3H, s), 1.37 (3H, s)]. 

**Table 1:** ^1^H-NMR and ^13^C-NMR spectral data of compound **1** (recorded at 600/150 MHz in CDCl_3_; δ in ppm, *J* in Hz).

Position	^13^C	^1^H (*J* in Hz)
1	45.4 (CH)	1.78 (m, 1H)
2	28.9 (CH_2_)	2.25 (m, 1H); 1.83 (ddd, *J* = 14.3, 7.4, 7.4 Hz, 1H)
3	130.3 (CH)	5.49 (dd, *J* = 7.4, 7.4 Hz, 1H)
4	128.1 (C)	
5	42.1 (CH_2_)	2.56 (dd, *J* = 14.4, 7.5 Hz, 1H); 2.29 (dd, *J* = 14.4, 3.0 Hz, 1H)
6	71.4 (CH)	4.91 (ddd, *J* = 8.0, 7.5, 3.0 Hz, 1H)
7	63.3 (CH)	3.04 (d, *J* = 8.0 Hz, 1H)
8	61.2 (C)	
9	38.3 (CH_2_)	2.04 (ddd, *J* = 13.4, 6.5, 3.0 Hz, 1H); 1.31 (ddd, *J* = 13.4, 13.4, 3.0 Hz, 1H)
10	23.6 (CH_2_)	2.24 (m, 1H); 1.98 (m, 1H)
11	125.1 (CH)	5.13 (dd, *J* = 7.0, 7.0 Hz, 1H)
12	135.0 (C)	
13	36.6 (CH_2_)	2.18 (dd, *J* = 11.2, 4.1 Hz, 1H); 2.14 (m, 1H)
14	28.5 (CH_2_)	1.74 (m, 1H); 1.27 (m, 1H)
15	85.6 (C)	
16	23.2 (CH_3_)	1.46 (s, 3H)
17	23.2 (CH_3_)	1.44 (s, 3H)
18	17.1 (CH_3_)	1.71 (s, 3H)
19	17.4 (CH_3_)	1.37 (s, 3H)
20	15.0 (CH_3_)	1.56 (s, 3H)
OAc	170.3 (C)	
	21.2 (CH_3_)	2.08 (s, 3H)
OAc	170.2 (C)	
	22.6 (CH_3_)	1.97 (s, 3H)

**Figure 2:**
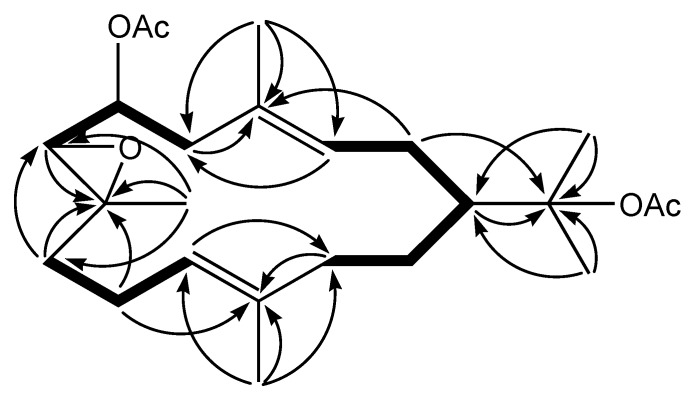
^1^H-^1^H COSY correlations (bold lines) and key HMBC correlations (H → C) of **1**.

In addition, the ^13^C-NMR spectra of **1** closely resembled those of **2** except for the presence of one additional acetoxy group. Therefore, it was suggested that compound **1** was a common 14-membered cyclic cembrane with an acetoxy, an acetoxyisopropyl and an epoxide functionalities. Assignments were performed based on ^1^H–^1^H COSY, HSQC and HMBC spectra data. ^1^H–^1^H COSY experiment revealed the sequences of the correlations depicted by the bold lines in Figure 2. 

In the HMBC experiment of **1**, the attachment of the acetoxyisopropyl group to C-1 was confirmed by correlations between H-1 to C-15, H-2 to C-15, H-16 to C-1 and H-17 to C-1. HMBC correlations between H-7 to C-8, H-9 to C-7 and C-8, H-10 to C-8 and H-19 to C-7, C-8 and C-9 were key for confirming that the methyl-bearing epoxide should be placed at the 7,8 positions. The vinyl methyl group at C-4 was confirmed by HMBC correlations between H-2 to C-4, H-5 to C-3 and C-4 and H-18 to C-3, C-4 and C-5. The other vinyl methyl group at C-12 was revealed by correlations between H-10 to C-12, H-11 to C-13, H-13 to C-12 and H-20 to C-11, C-12 and C-13. In addition, the chemical shift for C-6 (δ_c_ 71.4; δ_H _4.91) clearly indicated that the acetoxy group was attached to the oxymethine carbon at C-6. Based on these findings, the gross structure of **1** was determined to be as shown in Figure 1.

**Figure 3:**
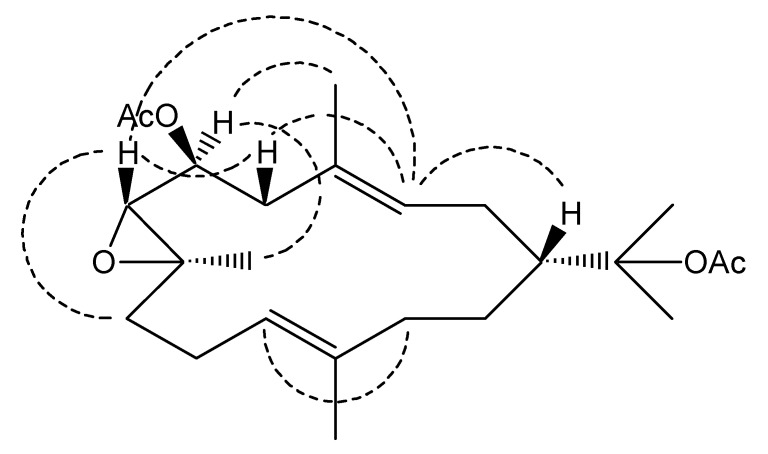
Key NOESY correlations of **1**.

The relative stereochemistry of compound **1** was deduced from the NOESY experiments (Figure 3), as well as the ^1^^3^C-NMR chemical shifts. The ^13^C-NMR chemical shifts of C-18 at δ_C_ 17.1 and C-20 at δ_C_ 15.0 suggested that both double bonds had the *E* configurations [3,10]. Furthermore, the NOESY correlations observed between H-3/H_2_-5 and H-11/H_2_-13 also supported this deduction. The NOESY correlations between H-1/H-3, H-3/H-5β, H-3/H-7 and H-5β/H-7 showed that these protons are oriented on the same side. In addition, the NOESY correlations between H-6/H_3_-18, H-6/H_3_-19 and H-7/H-9β but lack of correlation between H-7/H_3_-19 indicated that H-6 and H_3_-19 are located on the same face of the molecule, while H-7 was located on the opposite face. This was supported by the similarities of ^13^C NMR data between **1** and **2**, indicating the same relative configurations at C-1, C-7 and C-8. Therefore, compound **1** was identified as (3*E*,11*E*)-6,15-diacetoxy-7,8-epoxycembra-3,11-diene. Compound **2** is reported to have *R* configuration at C-1 [9]. A literature survey indicated that all cembrane diterpene isolated from the order Alcyonacea have 1*R* configuration [11]. The absolute configuration at C-1 of **1** may thus be deduced to be *R* from the biogenetic consideration and co-occurrence of compound **2** in the same specimen. All compounds were evaluated for antimicrobial activity against seven human pathogenic bacteria. Unfortunately, compounds **1** and **2** were inactive at 30 μg/disc.

## 3. Experimental

### 3.1. General

Optical rotations were measured on an AUTOPOL IV automatic polarimeter (Rudolph Research Analytical). ^1^H-NMR (600 MHz) and ^13^C-NMR (150 MHz) spectra were recorded with a JEOL ECA 600 instrument, with TMS as internal standard. HR-ESI-TOFMS spectrum was obtained with LCMS-IT-TOF (Shimadzu). Preparative TLC was performed with silica gel plates (Merck, Kieselgel 60 F_254_). Silica gel (Merck, Kieselgel 60, 70–230 mesh) was used for column chromatography. Analytical TLC was performed on Merck Kieselgel 60 F_254_. Spots were visualized by UV light or by spraying with a 5% phosphomolybdic acid-ethanol solution.

### 3.2. Biological material

The specimen of *Nephthea* sp. was collected from Layangan Island, Sabah (5º19’588’’N, 115º12’020’’E), on October 21, 2008. The gross morphological features of this soft coral were very similar to those of *Nephthea erecta.* The voucher specimen (MAR37789BOR) was deposited in the BORNEENSIS Collection of Institute for Tropical Biology and Conservation, Universiti Malaysia Sabah.

### 3.3. Extraction and isolation

The fresh soft coral (2.10 kg wet wt) was extracted with MeOH (5 L) at room temperature for 7 days. The crude extract was evaporated under reduced pressure and the residue was partitioned between EtOAc and H_2_O. The EtOAc fraction was further partitioned with hexane and 90% MeOH. The hexane fraction (1.20 g) was chromatographed on a Si gel column using hexane and EtOAc system of increasing polarity as eluant to yield four fractions. A portion of fraction 2 (24.5 mg) eluted with hexane/EtOAc (8:2) was submitted to repeated preparative TLC with CHCl_3_ and toluene to yield compounds **1** (4.4 mg) and **2** (2.2 mg).

### 3.4. 6-Acetoxy-7,8-epoxynephthenol acetate ***(1)***

Colorless oil; [α]:^25^_D_ –15.9 (*c* 0.39, CHCl_3_); HR-TOFMS *m*/*z*407.2778 [M+H]^+^ (calcd. for C_24_H_39_O_5_, 407.2792); ^1^H-NMR and ^13^C-NMR spectral data: see Table 1.

### 3.5. Epoxynephthenol acetate ***(2)***

Colorless oil; [α]:^25^_D_ –15.5 (c 0.22, CHCl_3_); ^13^C-NMR (CDCl_3_) δ: 170.3 (s, OCOCH_3_), 134.8 (s, C-12), 132.0 (s, C-4), 126.6 (d, C-3), 125.2 (d, C-11), 85.7 (s, C-15), 62.3 (d, C-7), 60.0 (s, C-8), 46.0 (d, C-1), 38.7 (t, C-9), 36.5 (t, C-5), 36.4 (t, C-13), 28.9 (t, C-2), 28.6 (t, C-14), 25.4 (t, C-6), 23.5 (t, C-10), 23.4 (q, C-16), 23.2 (q, C-17), 22.7 (q, OCOCH_3_), 16.9 (q, C-19), 15.8 (q, C-18), 15.0 (q, C-20).

### 3.6. Antibacterial bioassay

The antimicrobial bioassays for the isolated compounds were carried out using seven strains of human pathogenic bacteria: *Escherichia coli* (CSV01-08), *Proteus mirabilis *(CSV03-08), *Pseudomonas aurelis *(CSV04-08), *Salmonella enteridis *(CSV07-08), *Salmonella thyphymunium *(CSV08-08), *Staphylococcus aereus *(CSV09-08) and *Vibrio cholerae *(CSV10-08). The assay was performed as previously described [12].

## 4. Conclusions

As a part of our chemical investigation on Bornean soft corals, a new cembrane diterpene, 6-acetoxy-7,8-epoxynephthenol acetate (**1**) was isolated along with the known epoxynephthenol acetate (**2**), from a *Nephthea* sp. specimen collected from Layangan Island, Sabah. Their structures were established on the basis of spectral analysis. These findings have enriched our knowledge of the chemical constituents of Bornean soft corals. The isolated compounds **1** and **2** were tested for their antibacterial activities against human pathogenic bacteria by the disc diffusion method. Although both these compounds were found to have no activity, it is believed that they could pave the way to utilize secondary metabolites as chemotaxonomic markers for soft coral genus *Nephthea*.
